# Development of a Three-Dimensional Optical Verification Technology without Environmental Pollution for Metal Components with Different Surface Properties

**DOI:** 10.3390/ma15176139

**Published:** 2022-09-04

**Authors:** Chil-Chyuan Kuo, Zong-Yan He, Chil-Xian Lee

**Affiliations:** 1 Department of Mechanical Engineering, Ming Chi University of Technology, No. 84, Gungjuan Road, New Taipei City 243, Taiwan; 2 Research Center for Intelligent Medical Devices, Ming Chi University of Technology, No. 84, Gungjuan Road, New Taipei City 243, Taiwan; 3 Road Ahead Technologies Consultant Corporation, No.88, Sec. 1, Guangfu Rd., Sanchong Dist., New Taipei City 241, Taiwan

**Keywords:** environmental pollution, three-dimensional, optical measurement, matte coatings, average size error

## Abstract

Nowadays, the optical measuring approach is widely used in the precision machining industry due to high measurement efficiency. In the industry, measuring devices play a crucial role in the field of quality assurance. In practical engineering, the green measurement approach indeed plays an important role in the industry currently. In this study, a state-of-the-art green technique for three-dimensional (3D) optical measurements without environmental pollution is demonstrated, which is an environmentally friendly optical measurement method. This method can perform precise optical measurement without matte coatings. This work dealt with the possibility of measuring four metal components that were not sprayed with anything. The differences in the optical measurement results between with and without matte coatings were investigated and analyzed. It was found that the research result has practical value in the precision machining industry because average size errors of the four measurement objects with different surface properties can be controlled at about 3 µm, 0.1 µm, 0.5 µm, and 9 µm. A technical database with industrial value was established for optical measurements of metal components with different surface properties without matte coatings, which can serve as an alternative to the conventional 3D optical measurement.

## 1. Introduction

One of the biggest benefits of using metal products is the mechanical properties compared to plastic products. Metal additive manufacturing is a powerful technology to manufacture metal products with complex geometries across numerous industries. As the advantages of additive manufacturing have become more tangible, its applications have skyrocketed over the last few years. The optical measurement approach [1] is widely used in the ferrous or nonferrous metal industry due to the high measurement efficiency compared to contact measuring approach [2]. Wang et al. [3] proposed a unique structured light for quality control of a Ti alloy component fabricated by metal three-dimensional (3D) printing technology [4,5,6]. The proposed structured light improved the scanning and processing speed greatly. Rękas et al. [7] demonstrated a method to inspect the geometry of car body parts made by stamping using an optical 3D scanner. It was found that the proposed method speeds up the error correction process significantly. Affatato et al. [8] evaluated wear of mobile total knee polyethylene inserts using an optical 3D scanner. Results showed that percentage discrepancies of wear volumes and wear rates were in the order of about 10%. Affatato et al. [9] evaluated knee joint prostheses wear distribution using an optical 3D scanner. The technique has potential application for wear analysis in numerical simulation of other polyethylene joint replacement components. Valigi et al. [10] proposed a new approach to evaluate wear of biomedical devices and industrial components. It was found that the proposed method is simpler and faster with respect to other conventional wear evaluation approaches because the wear parameters can be extracted from the 3D map. In addition, the real distribution of the phenomenon of the different portions of the object is quantifiable and visible. Zhao et al. [11] used optical precision measurement for optimization of machining parameters in the ultra-precision machining of polar microstructures. Results showed that the optimized parameters can be determined for machining of polar microstructures using optical precision measurement. Kim and Yoon [12] characterized the thermal phenomenon using optical measurement. Results showed that the error was within 3.8% compared with the conventional measurement method. The proposed method is simple because it can be performed without the need of employing complex calibrations.

The 3D optical scanning method has some distinct advantages compared to the conventional contact measurement method, especially for measurement parts with complex geometry. Nowadays, a contactless optical 3D scanner named GOM ATOS Triple Scan II is widely used in the quality assurance department in various companies currently. This optical system uses blue light to carry out precise scans at high measurement speed. Sensors are designed for complex verification tasks in various applications. To achieve precise measurement results, the conventional approach in the contactless optical 3D measurement is that the measurement product is coated with an anti-glare mixture of titanium oxide (TiO_2_) powder [13] and ethanol by spraying gun. However, there are three distinct drawbacks of spraying a mixture on the measured object based on practical experience. Firstly, removing the sprayed mixture is a time-consuming process, especially as the measurement object has many fine structures. Secondly, the measurement object may be damaged during the removal process of the mixture. Finally, the spraying time of the mixture will be increased when the size of the measurement object is larger. 

To overcome the drawbacks of spraying mixture on the measurement object, an environmentally friendly 3D optical verification technology for metal components with different surface properties is proposed. The main objective of this work is to carry out precise 3D optical inspection of measurement objects without matte coatings. The feasibility experiment was performed with four metal components with different surface properties using eight measurement methods. The differences in the optical measurement results were analyzed. Finally, an empirical technical database for optical measurements of metal components with different surface properties without the use of spraying a mixture of ethanol and TiO_2_ powder was established.

## 2. Experimental Details

**Figure 1:**
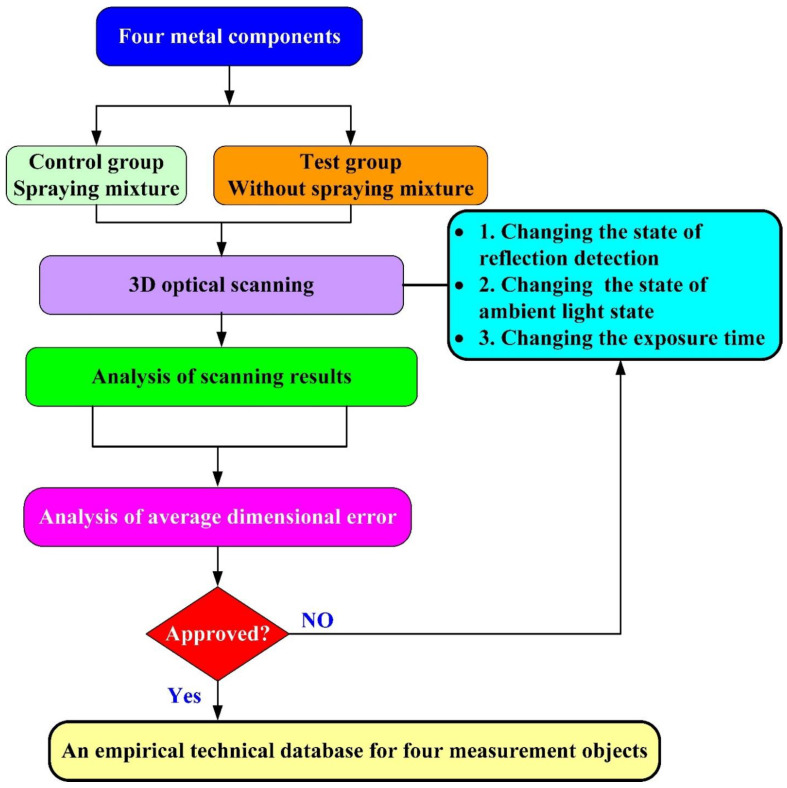
Research process of this study.

Figure 1 shows the research process of this study. In this study, four metal components were used to establish an empirical technical database. Table 1 shows the four metal components and their surface properties. The first measurement object is an as-cast turbine blade, which has a rough surface. The second measurement object is a cast part after computer numerical control (CNC) machining, which has a glossy surface. The third measurement object is a sheet metal, which has a glossy surface. The fourth measurement object is a circuit board, which has an uneven surface. The measurement data obtained by spraying the mixture on the measurement object surface is regarded as the control group. The measurement data obtained by not spraying anything on the surface of measurement object is considered as the test group. The full resolution, normal exposure time, and high quality of the scan were adjusted. Table 2 shows the eight measurement strategies proposed in this work. To prevent the sensor head from generating unnecessary reflected light, the sensor head was tilted at an angle of approximately 15° to 20°. The research point of experiment number one was to investigate the differences between more points and high quality in the optical measurement results for a measurement object that was not sprayed with anything. The research point of experiment number two was to investigate the differences between two exposure times and one exposure time. The research point of experiment number three was to investigate the effects of turning on reflection detection on the optical measurement results for a measurement object that was not sprayed with anything. The research point of experiment number four was to investigate the effects of turning on reflection detection under two exposure times on the optical measurement results for a measurement object that was not sprayed with anything. The research point of experiment number five was to investigate the differences in turning on or off ambient light. The research point of experiment number six was to investigate the differences in turning on or off ambient light. The research point of experiment number seven was to investigate the differences in turning on or off ambient light and reflection detection. The research point of experiment number eight was to investigate the differences in turning on or off ambient light and turning on reflection detection in terms of two exposure times.

**Figure 2:**
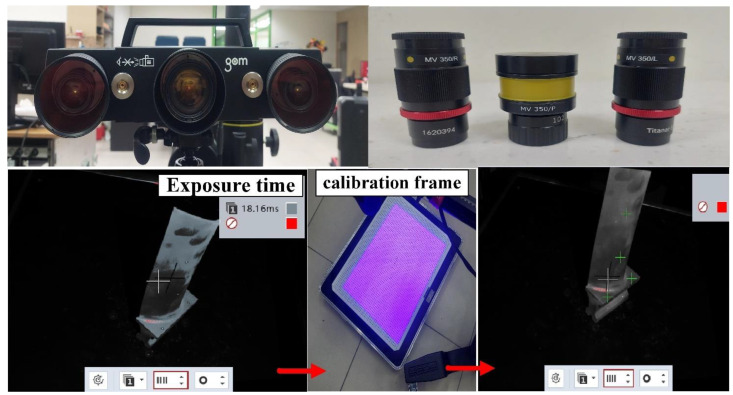
Calibration process before optical measurements.

**Figure 3:**
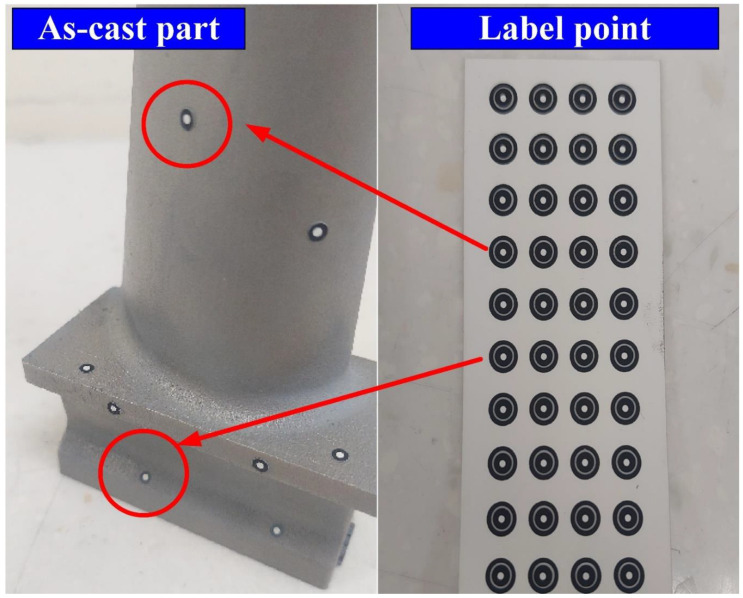
As-cast part after labeling the points.

The principle of 3D optical scanning is projecting particular patterns on the measurement object surface with a scanner and then capturing the images with cameras. The industrial 3D digitizing instruments (ATOS, GOM Inc., Essen, Braunschweig, Germany) with structure blue light source were used in this study [14,15]. This system includes one rotation stage (ROT 640) and two lenses (MV350 and MV500). The TiO_2_ powder with an average particle size of about 4 µm was selected as the main ingredient of the spraying mixture since it has a high refractive index of approximately 2.87. Therefore, total reflection can be formed during 3D optical measurement. The TiO_2_ powder and 95% ethanol were placed in the glass bottle placed in an ultrasonic machine for 10 min to prepare the mixture for matte coatings. The TiO_2_ powder and 95% ethanol were mixed in a weight ratio of 1:4 [16]. The speed of spraying was set to 15 cm/s. The spraying process parameters included a spraying angle of about 15° in combination with a spraying distance of about 10 cm. The quality of matte coatings was evaluated using an optical microscope (Quick Vision 404, Mitutoyo Inc., Tokyo, Japan). In general, calibration of the 3D digitizing instrument with a calibration frame was required before optical measurements. Figure 2 shows the calibration process before optical measurements. The exposure time was fixed at 1 s. Figure 3 shows the as-cast part after sticking on the reference points. The purpose of sticking reference points on the surfaces of the measurement objects is to make the scanning probe remember a relative position of the measurement object. Therefore, the scanned data can be superimposed using the number of the reference points. The stripe patterns were projected on the surfaces of measurement objects and captured by two cameras. Possible results can be obtained by applying GOM software. The scan data quality, resolution, scan area, and exposure can be adjusted in the software named GOM ATOS Professional V 7.5. Finally, a digital model of the measurement object in stereolithography (STL) format was exported. Thus, the coordinate measuring data can be determined from the projector and beam paths of both the cameras.

## 3. Results and Discussion

**Figure 4:**
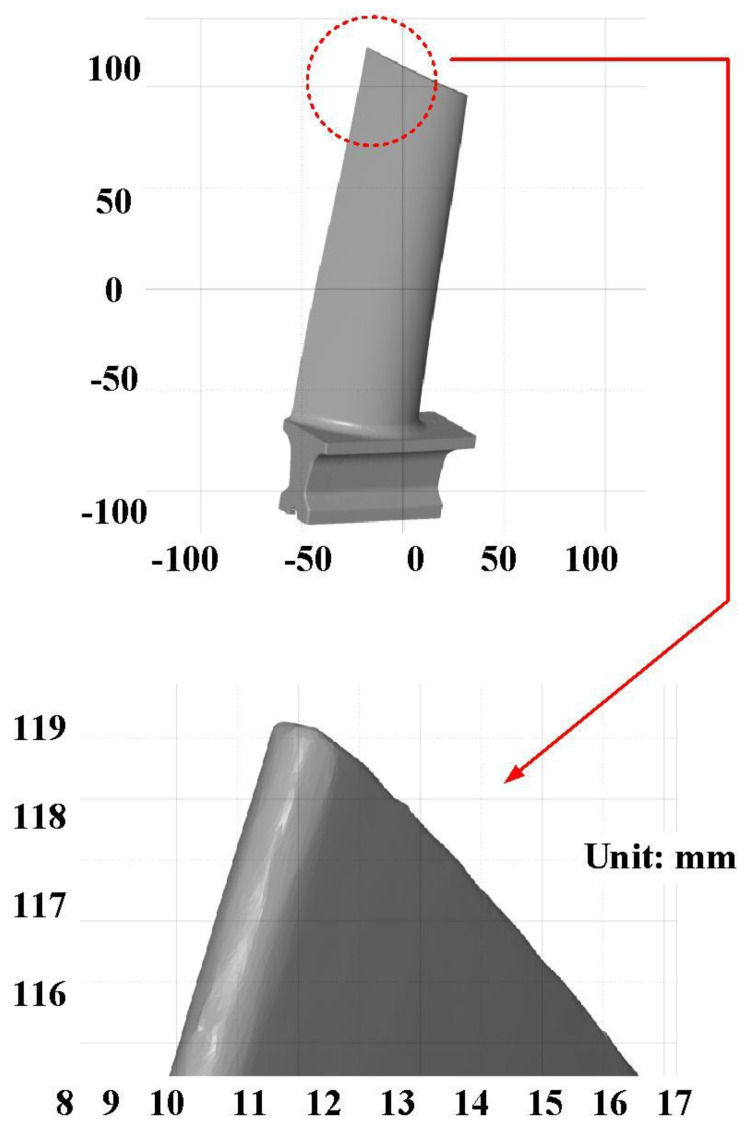
Measurement results of the as-cast turbine blade that was sprayed with a mixture of TiO_2_ powder and ethanol before optical measurements.

**Figure 5:**
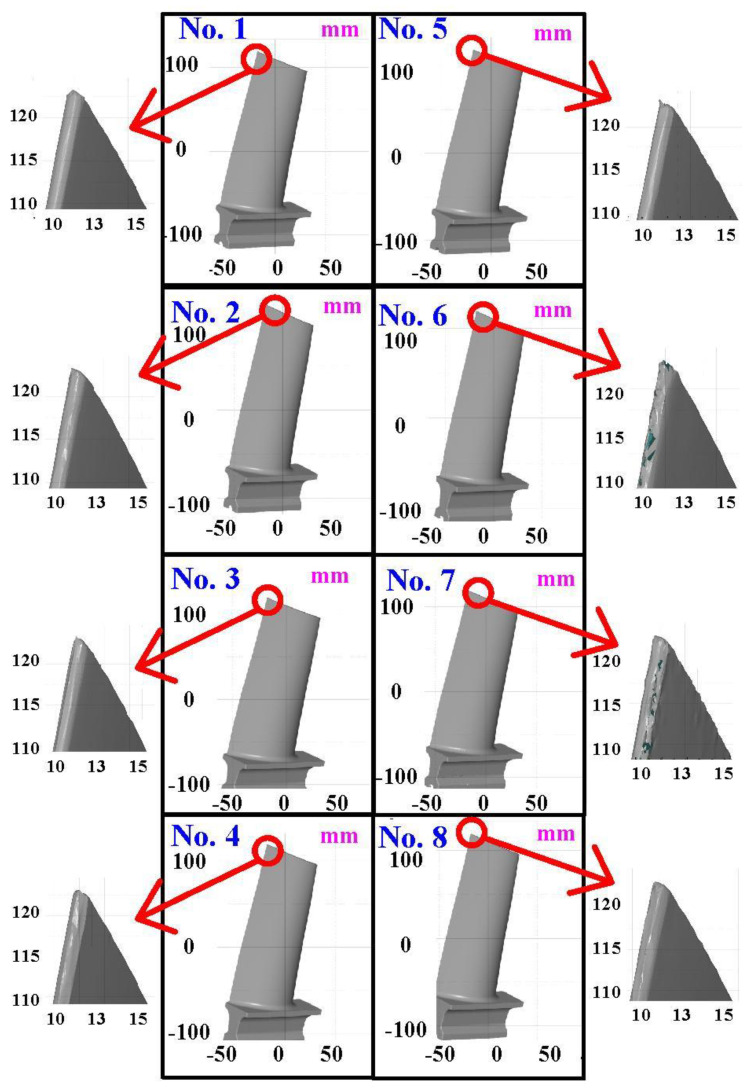
Measurement results of the as-cast turbine blade that was not sprayed with a mixture of ethanol and TiO_2_ powder.

**Figure 6:**
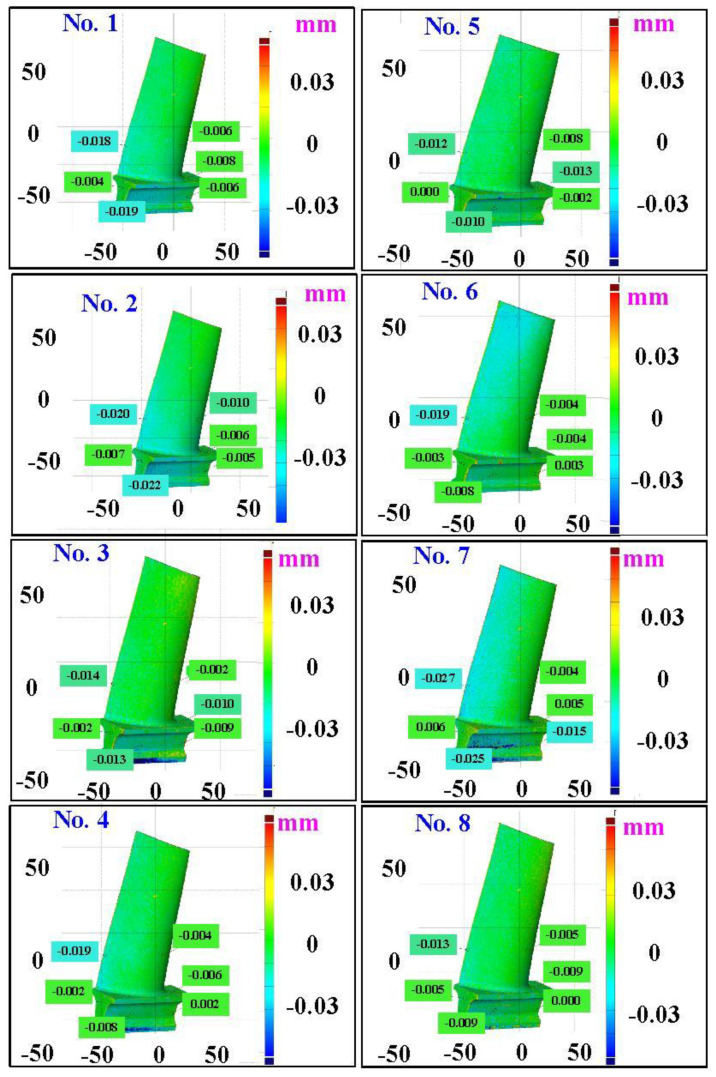
CAV results of the as-cast turbine blade.

**Figure 7:**
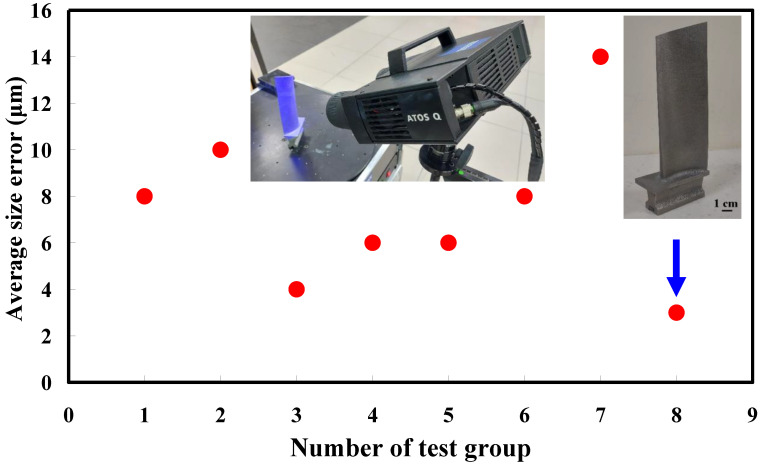
Average size error of the eight test groups compared to conventional method for the as-cast part.

The first measurement object is the as-cast turbine blade having a rough surface. This measurement object is made using the investment casting technique [17]. Figure 4 shows the measurement results of the as-cast turbine blade that was sprayed with a mixture of ethanol and TiO_2_ powder before optical measurements. The number of captured points is approximately 156,659 using the high quality function. Figure 5 shows the measurement results of the as-cast turbine blade that was not sprayed with a mixture of ethanol and TiO_2_ powder. The numbers of captured points for eight different measurement methods are approximately 218,781, 225,113, 212,619, 219,582, 217,377, 224,056, 222,458, and 220,494, respectively. The control group is considered as the nominal model. Figure 6 shows the computer-aided verification (CAV) results of the as-cast turbine blade. Note that the five measurement data shown in the figure are representative analysis results. The color deviation map represents the CAV results compared with the nominal model. Note that the incomplete geometry derived from data omission at the edge of the measurement object was the most serious for the test group numbers 6 and 7. Figure 7 shows average size error of the eight test groups compared to conventional method for the as-cast part. Compared to the overall measurement results from the control group, average size errors of the eight test groups are approximately 8 µm, 10 µm, 4 µm, 6 µm, 6 µm, 8 µm, 14 µm, and 3 µm, respectively. It is understandable that average size error of the 3D optical measurement parameters using number 8 of the test group is the lowest because average size error is only 3 µm. This means that the suggested measurement parameters for the as-cast turbine blade that was not sprayed with anything include two exposure times, more point, full resolution, scan all with reflection detection, and ambient light off.

**Figure 8:**
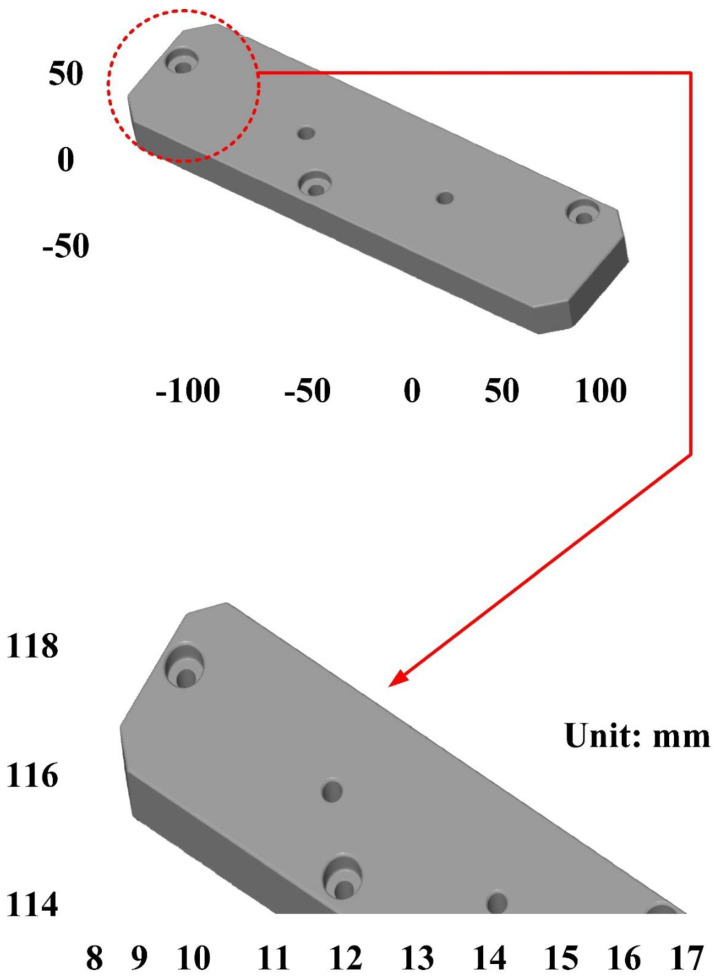
Measurement results of the cast part after CNC machining that was sprayed with a mixture of TiO_2_ powder and ethanol before 3D optical measurement.

**Figure 9:**
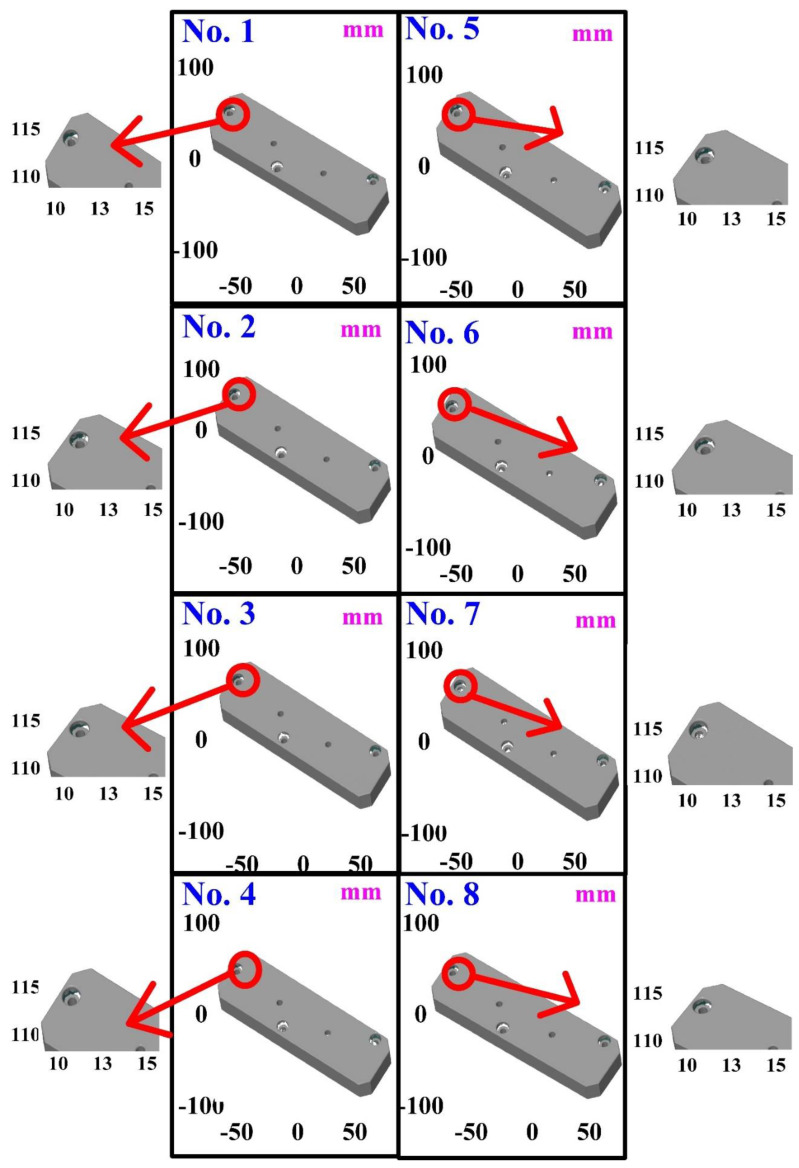
Measurement results of the cast part after CNC machining that was not sprayed with a mixture of ethanol and TiO_2_ powder.

**Figure 10:**
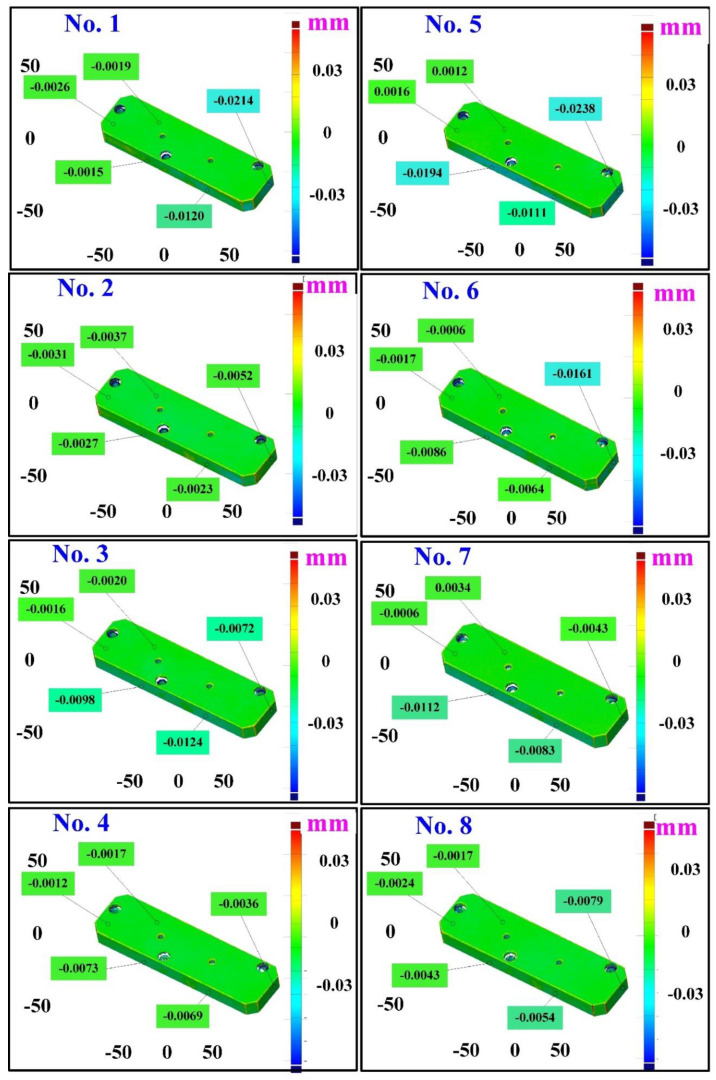
CAV results of the cast part after CNC machining.

**Figure 11:**
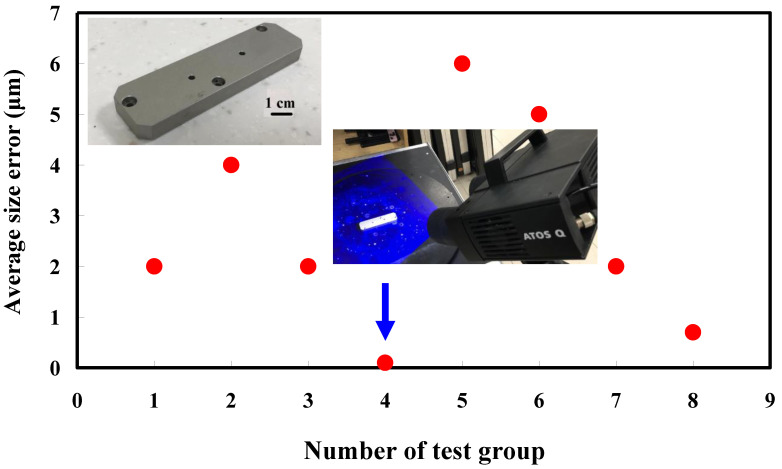
Average size error of the eight test groups compared to conventional method for the cast part after CNC machining.

The second measurement object is a cast part after CNC machining [18]. Especially, this measurement part has a glossy surface. The characteristic of a cast part after CNC machining is that the surface at the corners and edges of the object is relatively smooth. Thus, these places more easily produce reflections. Figure 8 shows the measurement results of the cast part after CNC machining that was sprayed with a mixture of TiO_2_ powder and ethanol before 3D optical measurement. The number of captured points is approximately 47,334 using the high quality function. Figure 9 shows the measurement results of the cast part after CNC machining that was not sprayed with a mixture of ethanol and TiO_2_ powder. The numbers of captured points for eight different measurement methods are approximately 89,991, 80,917, 86,748, 86,674, 99,382, 81,020, 85,846, and 77,427, respectively. The control group is considered as the nominal model. The color deviation map represents the CAV results compared with the nominal model. Figure 10 shows the CAV results of the cast part after CNC machining. Figure 11 shows average size error of the eight test groups compared to conventional method for the cast part after CNC machining. Compared to the overall measurement results from the control group, average size errors of the eight test groups are approximately 2 µm, 4 µm, 2 µm, 0.1 µm, 6 µm, 5 µm, 2 µm, and 0.7 µm, respectively. Note that the average size error of experiment number 4 and experiment number 8 of the test group is less than 1 µm. It is evident that the average size error of the 3D optical measurement parameters using number 4 of the test group is the lowest because average size error is only 0.1 µm. This means that the suggested measurement parameters for the as-cast turbine blade that was not sprayed with anything involve full resolution, two exposure times, more point, scan all with reflection detection, and ambient light on.

**Figure 12:**
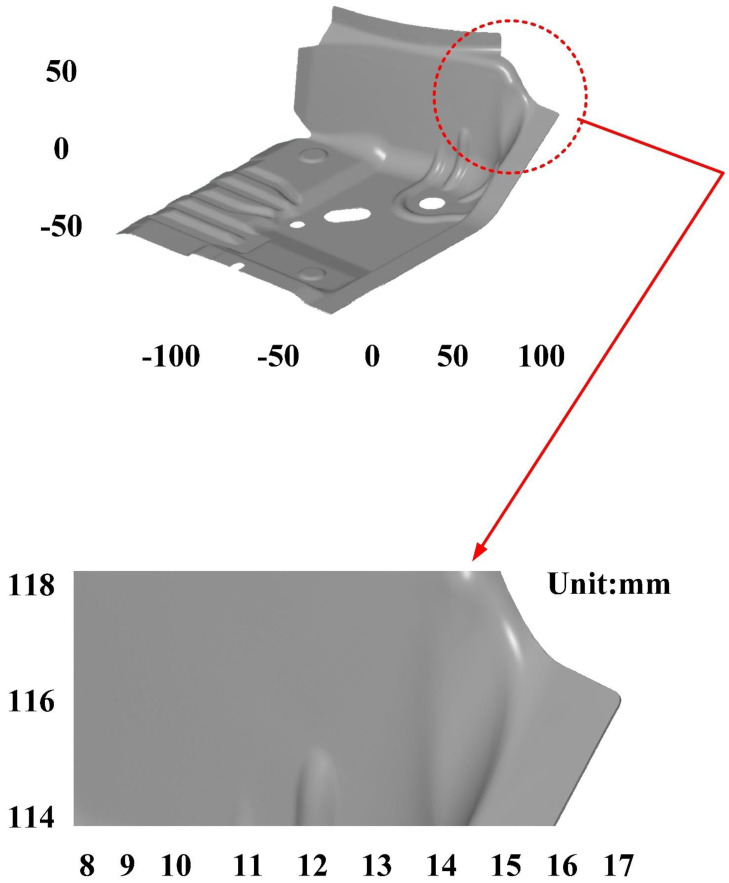
Measurement results of the glossy sheet metal that was sprayed with a mixture of TiO_2_ powder and ethanol before optical measurements.

**Figure 13:**
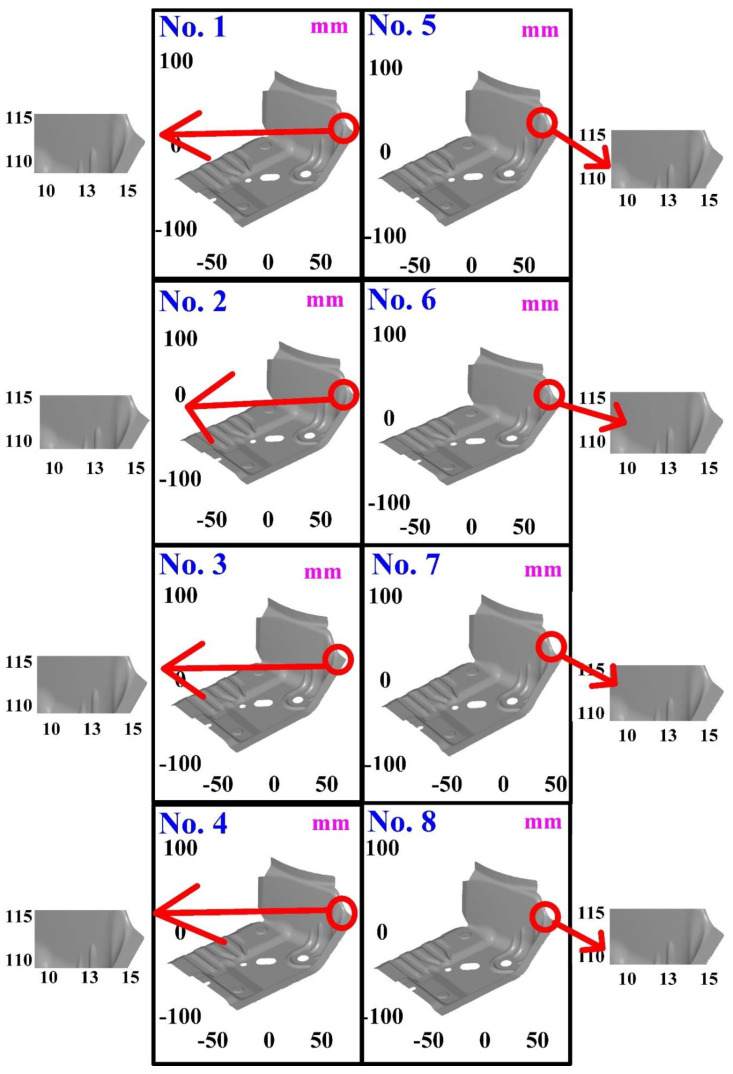
Measurement results of the glossy sheet metal that was not sprayed with a mixture of ethanol and TiO_2_ powder.

**Figure 14:**
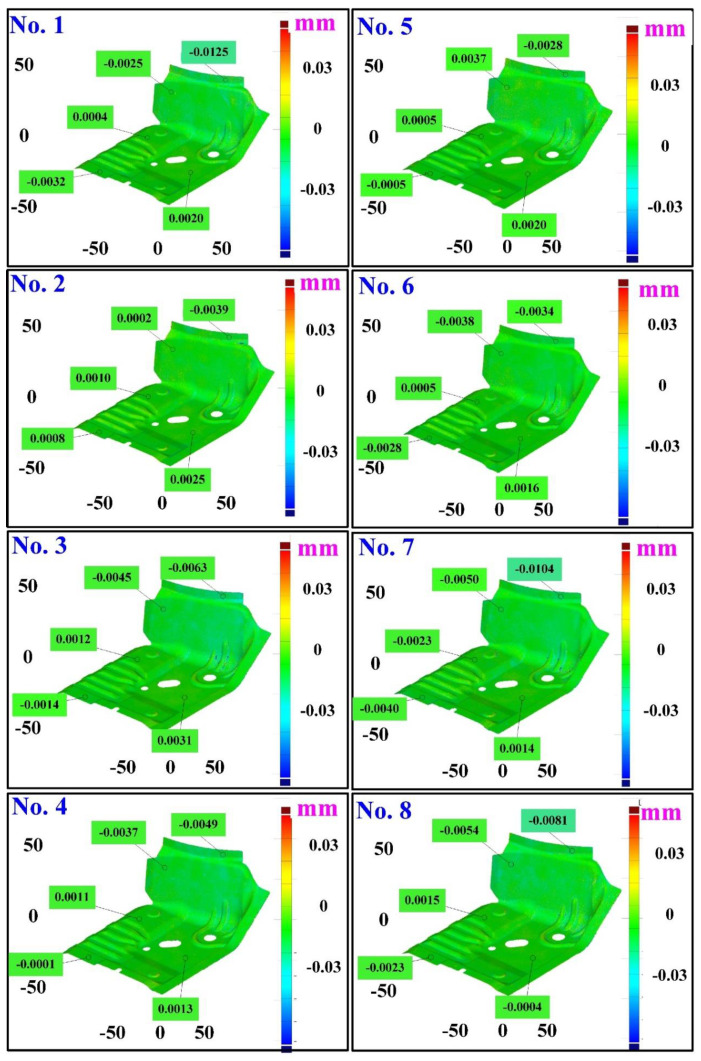
CAV results of the glossy sheet metal.

**Figure 15:**
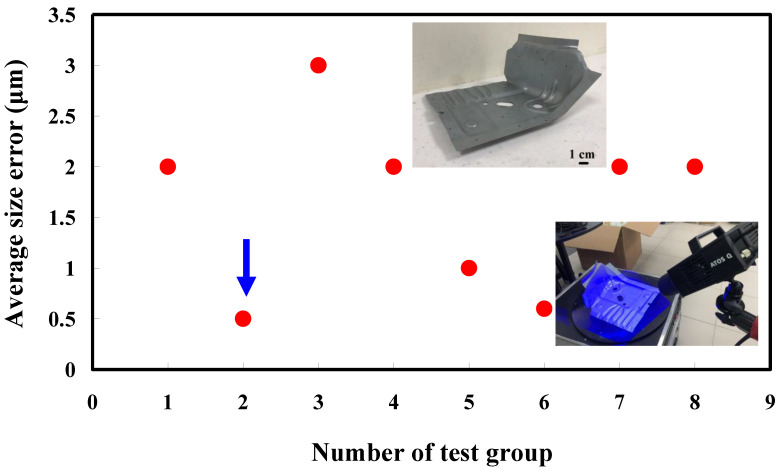
Average size error of the eight test groups compared to conventional method for the glossy sheet metal.

The third measurement object is a glossy sheet metal having a glossy surface. This measurement object is made using the stamping technique [19]. The characteristic of glossy sheet metal is that the surface of the object does not easily produce reflections. Thus, the reflections can be avoided through different measurement angles in the reflective areas. Figure 12 shows the measurement results of the glossy sheet metal that was sprayed with a mixture of TiO_2_ powder and ethanol before optical measurements. The number of captured points is about 1,792,675 using high quality function. Figure 13 shows the measurement results of the glossy sheet metal that was not sprayed with a mixture of ethanol and TiO_2_ powder. The numbers of captured points for eight different measurement methods are approximately 2,308,318, 2,573,995, 2,128,060, 1,922,071, 2,317,219, 2,056,374, 1,981,658, and 2,273,611, respectively. The control group is regarded as the nominal model. Figure 14 shows the CAV results of the glossy sheet metal. The color deviation map represents the CAV results compared with the nominal model. Note that the five measurement data shown in the figure are representative analysis results. Figure 15 shows average size error of the eight test groups compared to conventional method for the glossy sheet metal. Compared to the overall measurement results from the control group, average size errors of the eight test groups are approximately 2 µm, 0.5 µm, 3 µm, 2 µm, 1 µm, 0.6 µm, 2 µm, and 2 µm, respectively. The results showed that average size error is very small and the maximum average size error is only 3 µm. The main reason is that there are a few omissions in all the scanned data. In addition, the average size error of experiment number 2 and experiment number 6 of the test group is less than 1 µm. The main reason is that both experiment methods utilize two exposure times for 3D optical detection. The only difference between the two methods is that the ambient light was turned on in number 2 of the test group. It is obvious that the average size error of the 3D optical measurement parameters using number 2 of the test group is the lowest because the average size error is only 0.5 µm. This means that the suggested measurement parameters for the as-cast turbine blade that was not sprayed with anything involve full resolution, two exposure times, more point, scan all, and reflection detection on.

**Figure 16:**
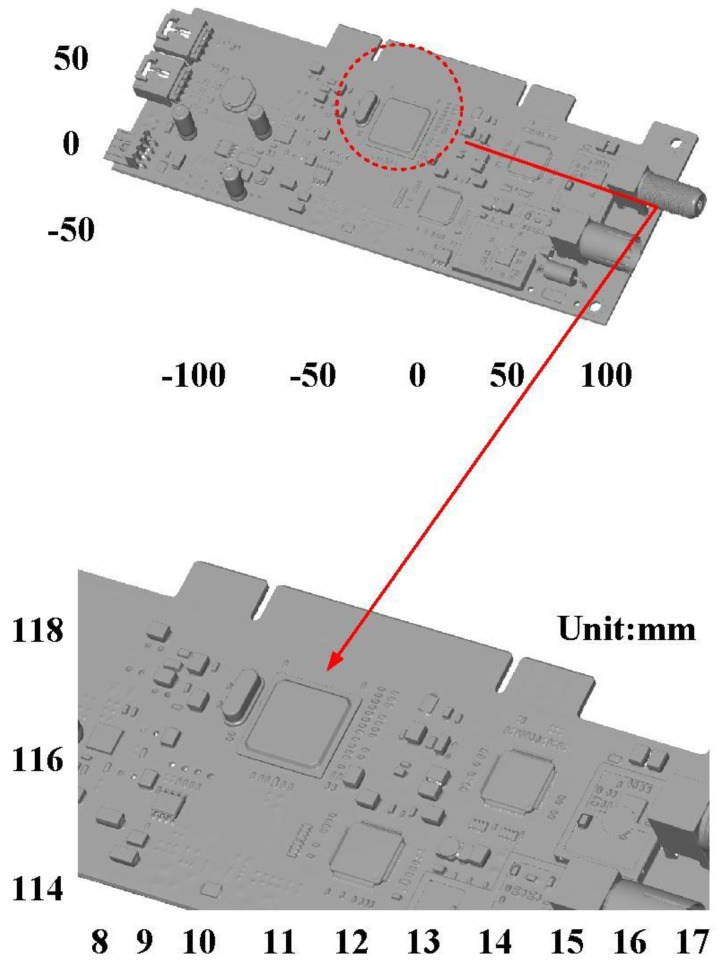
Measurement results of the circuit board that was sprayed with a mixture of TiO_2_ powder and ethanol before optical measurements.

**Figure 17:**
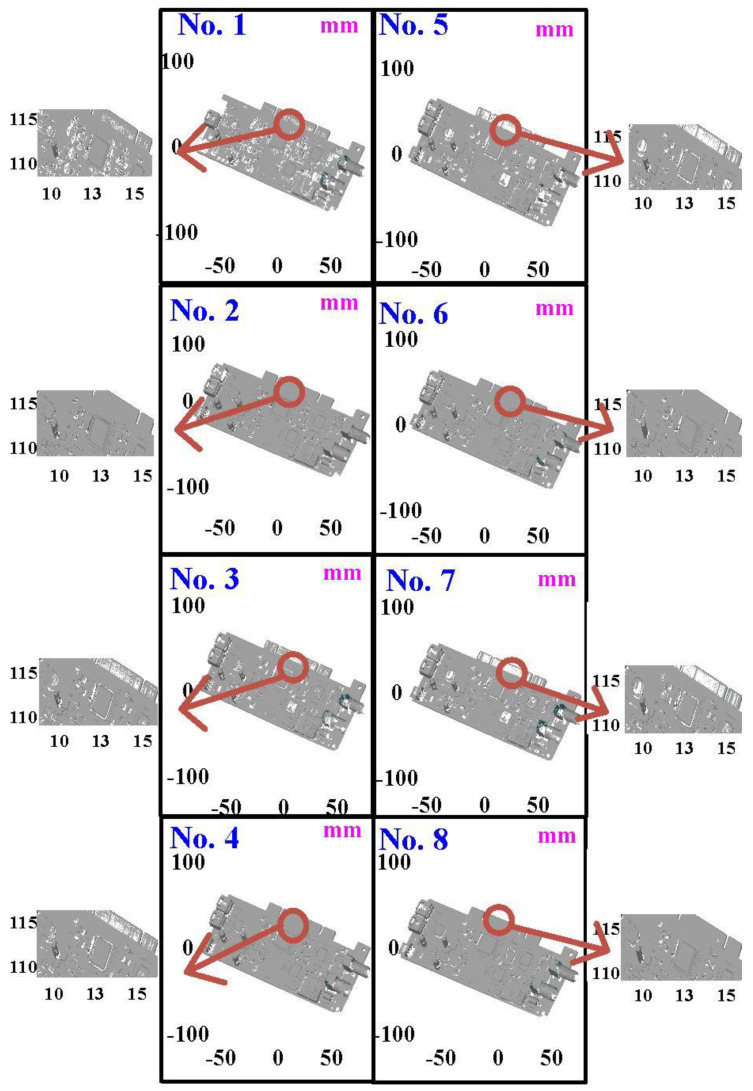
Measurement results of the circuit board that was not sprayed with a mixture of ethanol and TiO_2_ powder.

**Figure 18:**
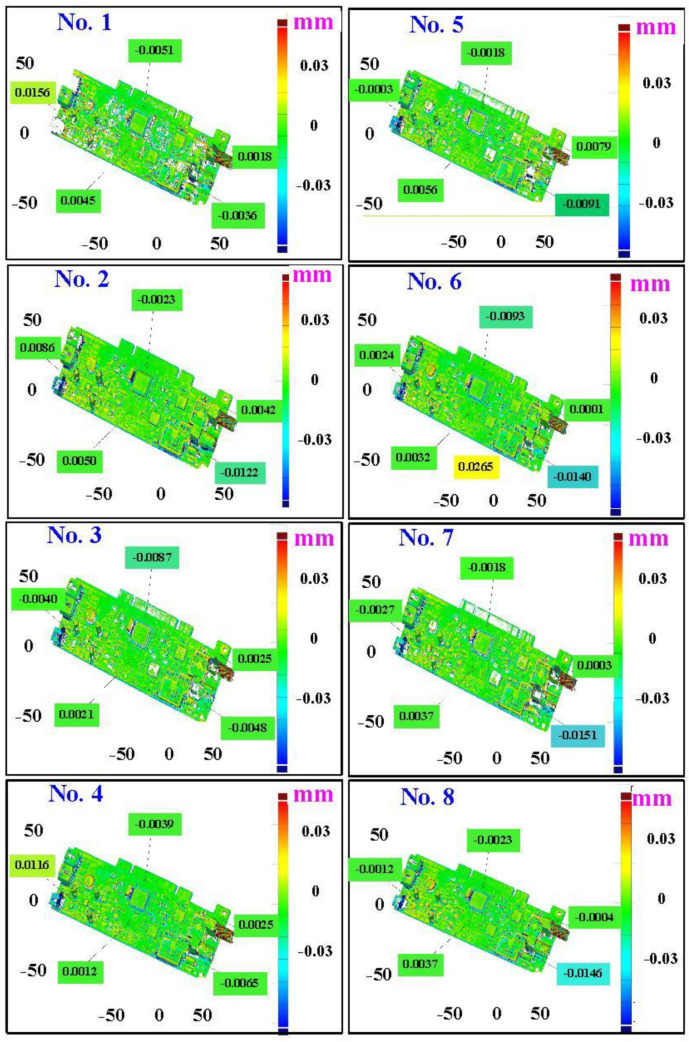
CAV results of the circuit board.

**Figure 19:**
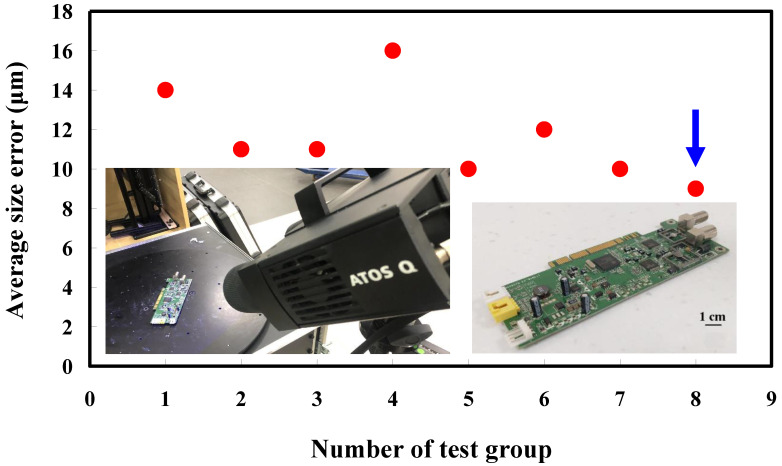
Average size error of the eight test groups compared to conventional method for the circuit board.

The fourth measurement object is a circuit board [20] having uneven surface. Especially, the circuit board has many reflections because there are many electronic components on the surface. Figure 16 shows the measurement results of the circuit board that was sprayed with a mixture of TiO_2_ powder and ethanol before optical measurements. The number of captured points is approximately 356,560 using the high quality function. Figure 17 shows the measurement results of the circuit board that was not sprayed with a mixture of ethanol and TiO_2_ powder. The numbers of captured points for eight different measurement methods are approximately 362,830, 384,853, 362,855, 370,602, 311,140, 329,956, 301,302, and 345,667, respectively. Figure 18 shows the CAV results of the circuit board. The color deviation map represents the CAV results compared with the nominal model. Note that the incomplete geometry derived from data omission at the edge of the measurement object was the most serious for the test group numbers 1, 3, 5, and 7. The main reason is that these four methods use one exposure time. The control group is considered as the nominal model and the color deviation map stands for the measurement results deviated from the nominal model. Figure 19 shows average size error of the eight test groups compared to conventional method for the circuit board. Compared to the overall measurement results from the control group, the average size errors of the eight test groups are approximately 14 µm, 11 µm, 11 µm, 16 µm, 10 µm, 12 µm, 10 µm, and 9 µm, respectively. It is obvious that the average size error of the 3D optical measurement parameters using number 8 of the test group is the lowest because the average size error is only 9 µm. This means that the suggested measurement parameters for the as-cast turbine blade that was not sprayed with anything involve full resolution, more point, two exposure times, scan all with reflection detection, and ambient light off.

According to the results described above, precise measurement of metal components that were not sprayed with anything using the suggested optical measurement parameters proposed in this study is possible. Table 3 shows an empirical technical database for 3D optical measurements of the object that was not sprayed with a mixture of ethanol and TiO_2_ powder. As can be seen, average size errors of the four measurement objects with different surface properties can be controlled for about 3 µm, 0.1 µm, 0.5 µm, and 9 µm. It is interesting to note that the detecting error is not affected by the size of the measurement objects. It should be noted that the detecting error is significantly affected by the surface property of the measurement objects. This empirical technical database can serve as an alternative to the conventional 3D optical measurements of the four metal components with different surface properties. It is interesting to note that the 3D optical measurement time is shortened greatly compared with conventional trial and error method. In addition, the total costs in the 3D optical measurements were reduced compared with conventional 3D optical measurements. Briefly, three distinct advantages were obtained from the environmentally friendly 3D optical verification technology proposed in this study: cost savings in the 3D optical measurements, savings in the lead time before optical measurements, and savings in the post-processing time of the measurement object after 3D optical measurements. Thus, the findings of this study can provide the greatest application potential in the precision machining industry for precise 3D optical measurements of the object that was not sprayed with a mixture of ethanol and TiO_2_ powder. Note that the environmentally friendly 3D optical verification technology proposed in this study is green technology [21,22,23] and meets the Sustainable Development Goals (SDGs) [24]. In this study, four metal components with different surface properties were used as measurement objects. It is important to note that the proposed environmentally friendly 3D optical verification technology can be employed for plastic products made of acrylonitrile butadiene styrene or polycarbonate [25,26,27,28,29]. In addition, the proposed environmentally friendly 3D optical verification technology can also be employed in the verification of feature sizes in the plastic injection mold [30], stamping die, blow molding mold [31], rotational molding mold, metal injection mold [32,33], or transfer molding mold [34]. In practical experience, three kinds of objects are difficult to scan. One is the transparent object [35], since the light cannot be reflected from the surfaces directly. Another is objects with ultimate shiny surface [36]. This is because too much light is reflected to the cameras. The other is dark objects [37], since the light reflected [38] to the cameras is not enough. These issues are currently being investigated and the results will be presented in a later work.

## 4. Conclusions

The contactless 3D optical scanning system is effective for measuring products with simple or complex geometry. The main purpose of this work was to propose an environmentally friendly 3D optical verification technology for metal components with different surface properties. The feasibility study was carried out with four metal components with different surface properties using eight different measurement approaches. Based on the results obtained in this study, the following conclusions can be drawn:

1. The remarkable findings in this study are very practical and provide the greatest application potential in the precision machining industry because the precise 3D optical measurements of the object without matte coatings were proven to work.
2. An empirical technical database for four measurement objects with different surfaces was built. This empirical technical database can serve as an alternative to the conventional 3D optical measurements because both the suggested approach and suggested measurement parameters are shown in Table 3. According to the information in Table 3, precise optical measurements without matte coatings for four similar metal components can be obtained. Average size error of the four different kinds of measurement objects of this characteristic can be controlled at 3 µm, 0.1 µm, 0.5 µm, and 9 µm.
3. The proposed environmentally friendly 3D optical verification technology meets the SDGs. Three advantages were obtained, i.e., cost savings in the 3D optical measurements, savings in the post-processing time of the measurement object after 3D optical measurements, and savings in the lead time before optical measurements.

## Figures and Tables

**Table 1:** Four metal components and their surface properties.

No.	Measurement Objects	Surface Property
1	As-cast turbine blade	Rough surface
2	Cast part after CNC machining	Glossy surface
3	Sheet metal	Glossy surface
4	Circuit board	Uneven surface

**Table 2:** Eight measurement strategies proposed in this work.

No.	Scan Area	Ambient Light	Exposure Time
1	Scan all	ON	One
2	Scan all	ON	Two
3	Scan all with reflection detection	ON	One
4	Scan all with reflection detection	ON	Two
5	Scan all	OFF	One
6	Scan all	OFF	Two
7	Reflection detection	OFF	One
8	Reflection detection	OFF	Two

**Table 3:** An empirical technical database for 3D optical measurement of the object that was not sprayed with a mixture of ethanol and TiO_2_ powder.

Measurement Product	Suggested Approach	Suggested Measurement Parameters	Average Size Error (µm)
As-cast turbine blade	8	Full resolution, more point, scan all with reflection detection, ambient light off, and two exposure times.	3
Cast part after CNC machining	4	Full resolution, more point, scan all with reflection detection, ambient light on, and two exposure times.	0.1
Glossy sheet metal	2	Full resolution, more point, scan all, reflection detection on, and two exposure times.	0.5
Circuit board	8	Full resolution, more point, scan all with reflection detection, ambient light off, and two exposure times.	9

## Data Availability

Not applicable.
